# Deep brain stimulation in the bed nucleus of the stria terminalis and medial forebrain bundle in a patient with major depressive disorder and anorexia nervosa

**DOI:** 10.1002/ccr3.856

**Published:** 2017-03-31

**Authors:** Patric Blomstedt, Matilda Naesström, Owe Bodlund

**Affiliations:** ^1^ Department of Pharmacology and Clinical NeuroscienceUmeå UniversityUmeåSweden; ^2^ Department of Clinical Sciences/PsychiatryUmeå UniversityUmeåSweden

**Keywords:** Anorexia nervosa, bed nucleus of the stria terminalis, deep brain stimulation, depression, medial forebrain bundle

## Abstract

Deep brain stimulation (DBS) may be considered in severe cases of therapy‐refractory major depressive disorder (MDD). However, DBS for MDD is still an experimental therapy. Therefore, it should only be administered in clinical studies driven by multidisciplinary teams, including surgeons with substantial experience of DBS in the treatment of other conditions.

## Introduction

In deep brain stimulation (DBS), thin quadripolar electrodes connected to a neuropacemaker are implanted into subcortical central structures of the brain where pathological neuronal activity is modulated with electrical current 1. The method has revolutionized the treatment of Parkinson′s disease and other movement disorders and is under investigation for, among others, some psychiatric conditions 2. In this group, DBS has shown some promising results, but the case material is limited and heterogeneous, consisting mainly of small nonrandomized studies with electrodes implanted in many different brain target structures 3. Here, we present a patient with severe major depressive disorder (MDD) and comorbid anorexia nervosa treated with DBS in the medial forebrain bundle (MFB) and subsequently in the bed nucleus of the stria terminalis (BNST). The MFB is typically regarded as a reward‐based pathway, and it is believed that dopaminergic neurotransmission plays an important role in MFB stimulation 4. The BNST serves as a major output pathway of the amygdala and has a complex role in regulating threat monitoring and anxiety. Dysfunction in this nucleus is believed to have an important role in anxiety disorders, partly through serotonergic activity 5.

## Case Presentation

### History

The patient, a 60‐year‐old woman, had a childhood onset of anxiety and anorexia nervosa, with symptoms of anxiety connected to food intake, restricted eating, and, later on, purging. The course of the eating disorder was remitting and relapsing with episodes at age 14, 28, and, most recently, age 44. The last episode had a prolonged course and, over time her, depressive symptoms became more and more severe, and since the age of 47, her main problem was MDD, with significant symptoms of anxiety. By the end, her eating disorder had clear depressive components with thoughts of being a burden on relatives, of eating being worthless and having suicidal ideations about starving to death. At the age of 54, the patient was committed to a closed psychiatric ward.

### Treatment

The patient had tried and failed psychotherapy, including several different classes of antidepressant, for example, selective serotonin reuptake inhibitors, monoamine oxidase inhibitors (MAOIs), tricyclic antidepressants, mood stabilizers, neuroleptics, ketamine infusions, and transcranial magnetic stimulation with little or no effect (details of medication trials are presented in detail in Table 1). The only treatment providing relief was electroconvulsive therapy (ECT), and since many years ago, she had had three sessions of ECT every 4 weeks. Unfortunately, ECT resulted in a gradual loss of memory, finally removing most of her memories from before her 30th year of age. Attempts to reduce the frequency of ECT sessions failed as this resulted in several suicide attempts while being treated.

**Table 1:** Previous medications administered with dosing regimens and length of medication trials

Medication	Dosage regime	Duration
Phenelzine	15 mg 2 + 1 + 1	6 weeks
Valproate	500 mg 1 + 0 + 1	4 weeks
Lithium	42 mg 1 + 0 + 1	102 weeks
Pregabalin	150 mg 1 + 0 + 1	39 weeks
Perphenazine	2 mg 0 + 0 + 1	84 weeks
Olanzapine	2.5 mg 1 + 0 + 0	30 weeks
Clomipramine	75 mg 0 + 0 + 2	153 weeks
Escitalopram	20 mg 1 + 0 + 0	78 weeks
Moclobemide	300 mg 1 + 0 + 1	20 weeks
Bupropion	150 mg 1 + 0 + 0	5 weeks
Duloxetine	60 mg 0 + 1 + 0	32 weeks
Sertraline	50 mg 1 + 0 + 0	4 weeks
Mirtazapine	15 mg	Discontinued due to drug‐induced granulocytopenia

Therefore, after extensive screening and obtaining her informed consent, the patient was included in an ongoing study of DBS for major depressive disorder (MDD). The MFB was chosen as the target based on a recent report, which highlighted the acute and quick effect of this treatment 6.

When she was evaluated before surgery at baseline, the patient weighted 40 kg with a body mass index (BMI) of 16.6. She was deemed to be severely depressed. She scored 43 points on the Montgomery–Asberg Depression Rating Scale (MADRS), 22 on the Hamilton Rating Scale for Depression (HAM‐D), and 34 on the Hamilton Rating Scale for Anxiety (HAM‐A). She preferred lying alone in a dark room. She exhibited reduced facial mimicry. She responded adequately to questions, but with short sentences and a monotonous voice.

**Figure 1:**
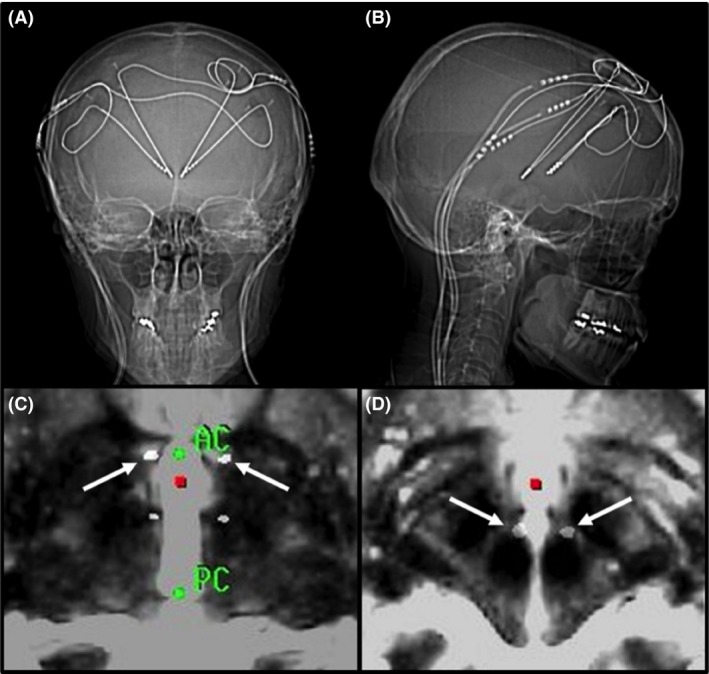
Postoperative scans demonstrating the location of the electrodes: (A) CT coronal view; (B) CT sagittal view; (C) CT fused with T2 MRI at AC‐PC‐level with arrows indicating the electrodes in the BNST; (D) CT fused with T2 MRI 4 mm below AC‐PC‐level with arrows indicating the electrodes in the MFB. CT, computed tomography; MRI, magnetic resonance imaging; AC, anterior commissure; PC, posterior commissure; BNST, bed nucleus of stria terminalis; MFB, medial forebrain bundle.

At age 56, the patient underwent implantation of two DBS electrodes (Medtronic model 3389) in the area of the MFB in the posterior hypothalamic area, just anterior to the red nucleus (Fig. 1). Stimulation was initiated 2 days after surgery. When the patient returned 1 week later, the effect was perceived as being dramatic. She considered herself to be “quite happy.” She had a normal facial mimicry, spoke fluently, and smiled occasionally.

Bipolar stimulation was delivered using three contacts on each side, at 130 Hz and 60 *μ*sec. The voltage was gradually increased and after 4 months was 2.8 V on the left side and 3.0 V on the right. A further increase was not possible as it caused blurred vision. At 6 months, MADRS was reduced to 26 points, HAM‐D 22 and HAM‐A to 21 (results are presented in detail in Table 2).

**Table 2:** Evaluations before and 6, 12 and 24 months after first surgery, and 6 and 12 months after second surgery

	Baseline pre‐op	6 months	12 month	24 months/Baseline second surgery	(30 months) 6 months	(36 months) 12 months
MADRS	43	26	33	27	14	13
HAM‐D	22	22	30	15	15	6
HAM‐A	34	21	24	27	10	15
GAF	30	45	52	55	60	65
BMI	16.2	16.2	16.5	15.2	14.5	14.3
NRS‐11						
Depression	NA	7	8	8	1	0
Anxiety	NA	4	5	3	1	0
Obsessions	NA	8	6	1	0	0
Compulsions	NA	1	2	1	0	0

MADRS, Montgomery–Asberg Depression Rating Scale; HAM‐D, Hamilton Rating Scale for Depression; HAM‐A, Hamilton Rating Scale for Anxiety; GAF, global assessment of function; BMI, body mass index; NRS‐11, numeric rating scale 0–10; NA, not available.

Ten months after the procedure, the patient complained of blurred vision. Numerous adjustments of stimulation and the cessation of lamotrigine (given the possibility of the drug being able to attenuate visual side effects) were tried in the following period without success 7. The symptom was partly stimulation induced, but even after the stimulation had been turned off for 2 weeks, some minor symptoms remained. The stimulation was restarted with a voltage reduced to a level where the side effects were tolerable, however, with a reduced effect on her psychiatric symptoms.

Two years after the first procedure, the patient was therefore re‐operated upon, with implantation of bilateral electrodes (Medtronic model 3387) in the BNST (Fig. 1). The patient received monopolar stimulation through two contacts on each electrode with a gradually increasing voltage. At 12 months, the patient received 130 Hz, 120 *μ*sec and 4.3 V bilaterally. The stimulation in the MFB was reduced and turned off simultaneously without any signs of deterioration.

### Outcome and follow‐up

The improvement seen after BNST DBS was more gradual, but very profound. Nine months after surgery, the patient was released from the psychiatric ward and returned to her home. Prior to this, she had been subject to hospital care, initially, due to her eating disorder and lastly due to severe MDD with suicidal ideation, for almost 4 years. She is now living full time at home with her family and is participating in social gatherings and outdoor activities. She considers herself to be profoundly improved, and at 12 months, her MADRS was reduced to 13 points, with an HAM‐D score of six and an HAM‐A of 5 points.

Throughout the postoperative periods, neither of the surgical procedures had any significant effect on her anorexia, in terms of BMI. However, following the second procedure, all her anxiety concerning food and eating vanished. She has virtually stopped vomiting, her food intake is more stable and less prone to large variations, and tube feeding could be discontinued. However, in the words of the patient, she continues, out of habit to eat just enough to keep her weight stable, even in the absence of anxiety or obsessive thoughts. She is now, however, motivated to start behavioral training to change this pattern.

## Discussion

According to WHO, depression is one of the most common cause of disability with a prevalence of 3–5% and the STAR*D studies have demonstrated the limitations of conventional treatments 8, 9, 10. Not only is depression associated with the suffering of the patients, an often severe social handicap and a reduced quality of life, but also with a significant mortality. It is estimated that 90% of the suicides are related to psychiatric diseases, the most common cause being depression, where the mortality due to suicide is around 10–15% 11. The frequence of therapy‐resistant MDD is estimated to range from 12% to −30% 12, 13.

Anorexia nervosa has one of the highest mortality rates of any psychiatric disorder, and the presence of anxiety and mood disturbances portends a worse prognosis of the disorder 14. As for depression, conventional treatment methods have demonstrated limitations. Pharmacological methods have been shown to be ineffective in anorexia nervosa 15. Even with psychotherapy and self‐help programs, with an effectiveness of around 50%, there remains a group of patients with intractable symptoms 16.

Even though the majority of patients will respond well to noninterventional therapy, there remains a significant group in both depression and anorexia nervosa, in whom conventional treatment will yield little or no relief of symptoms. In severely affected patients in whom therapy‐resistant symptoms have caused a high degree of suffering and handicap, interventional procedures in the form of stereotactic functional neurosurgery might be indicated.

The experience of DBS in MDD is still limited. A total of 100 patients treated with DBS for MDD in nine different studies and involving several brain targets have been published 17. The most common targets are the subcallosal cingulate gyrus (SCCG), the nucleus accumbens (NA), and the ventral caudate/ventral striatum (VC/VS) 18, 19, 20. The inferior thalamic peduncle and the lateral habenula were the target in two case reports 21, 22. The most recently published brain target for DBS in depression is the MFB, where results have been presented for seven patients 6. The results of DBS for depression have generally been promising, although recent blinded randomized multicenter studies in the USA have failed to demonstrate a benefit of active stimulation compared to sham stimulation 19. Regarding the BNST, reports on this target have only been published in one study on DBS for obsessive compulsive disorder (OCD), but not on MDD 23.

In our patient, the MFB, connecting the amygdala, ventral tegmental area, the NA, ventromedial, and the lateral nuclei of the hypothalamus, was initially chosen as the target as the onset of effect has been reported to be rapid 6. A fast onset of effect was deemed to be essential considering the patient's dependency on ECT and the fact that it may not be possible to administer ECT after DBS. Blurred vision following this procedure was described in the original publication 6, even though not in the manner described here with a late appearance and semireversibility.

When a second surgery was considered to be indicated, the BNST, a part of the anxiety‐regulating network between the amygdala, hypothalamus, thalamus, and the orbitofrontal cortex 23, was chosen as the target. This decision was based on our own experience of the effect of BNST DBS for concomitant depressive symptoms and anxiety in patients with OCD and generalized anxiety disorder (GAD) (unpublished data). Furthermore, studies have pointed out the BNST as an important brain structure involved in anorexia nervosa and anxiety disorders 23, 24, 25, 26.

Even though the indication for this procedure was MDD, it would not have been unreasonable to expect a positive effect on the patient's concomitant anorexia. The effects of improved mood and anxiety could potentially disrupt important illness‐maintaining factors. The improvement in this patient's mood, anxiety, and quality of life, despite the remaining sign of underweight, is promising, in view of the well known poor response of underweight patients to conventional pharmacological and psychological therapies. In the literature published in five reports, DBS for anorexia has been performed on three different brain targets in 14 patients, several with concomitant MDD, OCD, or GAD. The SCCG was targeted in seven patients 27, 28, NA in six 29, 30, and the VC/VS in one 31. Most patients in this heterogenous material seem to have benefited to various extents from the procedures.

## Conclusion

Even though DBS might offer hope to patients with severe treatment‐resistant MDD, it is important to stress that DBS for MDD is a still an experimental therapy. Therefore, prior to labeling a case of MDD as “treatment‐resistant,” it is essential to ensure that adequate trials of treatment methods have been conducted. There is currently no consensus on the definition of treatment‐resistant MDD, and clinical trials differ in inclusion criteria 32. Prior to considering referral for such experimental therapies as DBS for MDD, we suggest that practicing clinicians should follow local guidelines for MDD treatment, including considering the use of more aggressive antidepressant treatments, such as ECT and MAOIs.

## Authorship

PB and MN: conducted the acquisition of information for the case and drafted the initial version of the manuscript. OB: critically edited and revised the initial draft of the manuscript with regard to important intellectual content, with a focus on the psychiatric aspects. All authors discussed the case and commented on the manuscript at all stages and gave their final approval of the version to be published in Clinical Case Reports.

## Conflict of Interest

PB: is a consultant for Medtronic and a shareholder in Mithridaticum AB. MN: has no disclosure or conflict of interest to declare. OB: has no disclosure or conflict of interest to declare.
